# Biological Pathways Associated with Neuroprogression in Bipolar Disorder

**DOI:** 10.3390/brainsci11020228

**Published:** 2021-02-12

**Authors:** Bianca Wollenhaupt-Aguiar, Flavio Kapczinski, Bianca Pfaffenseller

**Affiliations:** 1 Department of Psychiatry and Behavioural Neuroscience, McMaster University, Hamilton, ON L8N 3K7, Canada; wollenhb@mcmaster.ca (B.W.-A.); kapczinf@mcmaster.ca (F.K.); 2 Mood Disorders Program, St. Joseph’s Healthcare Hamilton, Hamilton, ON L8N 3K7, Canada; 3 Neuroscience Graduate Program, McMaster University, Hamilton, ON L8S 4L8, Canada; 4 Instituto Nacional de Ciência e Tecnologia Translacional em Medicina (INCT-TM), Universidade Federal do Rio Grande do Sul (UFRGS), Porto Alegre 90035-903, Brazil; 5 Department of Psychiatry, Universidade Federal do Rio Grande do Sul (UFRGS), Porto Alegre 90035-003, Brazil

**Keywords:** biomarkers, bipolar disorder, illness progression, neuroprogression

## Abstract

There is evidence suggesting clinical progression in a subset of patients with bipolar disorder (BD). This progression is associated with worse clinical outcomes and biological changes. Molecular pathways and biological markers of clinical progression have been identified and may explain the progressive changes associated with this disorder. The biological basis for clinical progression in BD is called neuroprogression. We propose that the following intertwined pathways provide the biological basis of neuroprogression: inflammation, oxidative stress, impaired calcium signaling, endoplasmic reticulum and mitochondrial dysfunction, and impaired neuroplasticity and cellular resilience. The nonlinear interaction of these pathways may worsen clinical outcomes, cognition, and functioning. Understanding neuroprogression in BD is crucial for identifying novel therapeutic targets, preventing illness progression, and ultimately promoting better outcomes.

## 1. Introduction

Bipolar disorder (BD) affects approximately 60 million people worldwide and negatively impacts their quality of life, functioning, and overall health from a young age [1]. Furthermore, BD is among the top 20 disorders with the highest global burden of disease, and patients with BD have a reduced life expectancy of about 8–12 years [2]. In addition, a recent study showed that functional impairment is progressive in a subset of patients, characterized primarily by a higher number of relapses, greater neurocognitive impairment, and greater overall severity of depressive symptoms [3], which corroborates previous studies showing that patients who had multiple episodes present with cognitive and functional impairment [4,5]. A recent meta-analysis also showed that the risk of progression to dementia is higher in patients with BD [6]. In addition, the number of mood episodes predicts the development of dementia in BD [6].

Furthermore, recent studies have shown that multiple mood episodes are associated with brain changes in BD [7]. For instance, brain imaging showed that reductions in hippocampal subfields [8], total hippocampal volume [9], brain regions covering the fronto-limbic system (gray matter), cerebellum, and corpus callosum (white matter) [10] are associated with a higher number of mood episodes. Other studies have also reported associations between impairments in verbal learning memory and a higher number of mood episodes [9].

**Figure 1:**
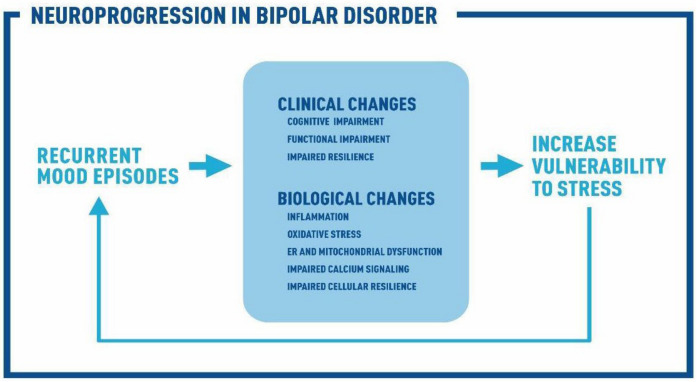
Neuroprogression in bipolar disorder (BD). Association between clinical changes (cognitive and functional impairment and impaired resilience), biological changes (inflammation, oxidative stress, impaired calcium signaling, endoplasmic reticulum (ER) and mitochondrial dysfunction, impaired cellular resilience) and recurrent mood episodes in the clinical progression of BD. As the illness progresses, the clinical and biological changes associated with mood episodes become more pronounced, increasing the patient’s vulnerability to stress and new episodes which in turn reinforces the cycle of neuroprogression in BD.

The impact of repeated mood episodes in clinical outcomes over time is known as clinical progression [11]. The biological basis for clinical progression in BD is called neuroprogression [12]. Importantly, cognitive impairments in BD have been linked with low-grade inflammation and brain structural alterations [13]. As the illness progresses, the clinical and biological changes associated with mood episodes become more pronounced, increasing the patient’s vulnerability to stress and new episodes which in turn reinforces the cycle of neuroprogression in BD (Figure 1).

In past years, research has focused on peripheral biomarkers as a way of understanding the underlying basis of BD [14]. Studies showed that patients at a late stage of BD presented decreased serum levels of brain-derived neurotrophic factor [15] and increased levels of inflammatory markers such as tumor necrosis factor-alpha (TNF-alpha) [15,16] and interleukin (IL)-6 [17], as well as the C-C motif ligand 11 (CCL11) [18]. Changes in peripheral biomarkers were also shown in meta-analyses [19,20,21]. The development of biomarkers to improve diagnosis, treatment, and prognosis is an ongoing pursuit in the field of BD. Due to the heterogeneity of BD, the possibility of developing a single, specific biomarker is still remote; however, there is a set of promising biomarkers that may serve as predictive, prognostic, or diagnostic markers in the future. Recently, a composite biomarker panel was able to discriminate between patients with rapid-cycling BD and healthy individuals and between manic and depressive states [22]. It was also shown that machine learning techniques, coupled with peripheral biomarkers, provided a potential diagnostic tool to aid in distinguishing depressed patients with BD from major depressive disorder [23]. The field has been investigating several biological pathways, mostly associated with inflammation, oxidative stress, neurotrophic factors, and cellular resilience. Based on the available evidence, in this overview, we suggest a potential mechanistic association between these pathways and the course of the illness in BD.

## 2. Inflammation

There is increasing evidence suggesting that chronic inflammatory processes in the periphery and in the brain (neuroinflammation) are involved in the pathophysiology of BD [24]. Low-grade immune activation has been reported in BD, related to the mood states [25,26], illness progression [15,18], and higher rates of comorbidities, including metabolic syndrome, cardiovascular, and cerebrovascular disease [27,28]. Several meta-analyses have shown increased levels of inflammatory markers in patients with BD, including soluble interleukin (IL)-2 receptor (sIL-2R), sIL-6R, TNF-alpha, soluble TNF receptor-1, and IL-4 [19,20,21,22]. This immune response may be generated and amplified by the actions of the damage-associated molecular patterns (DAMPs); some of them—circulating cell-free (ccf) nuclear (n)DNA, heat shock protein (HSP)70, and HSP90a—were found to be increased during BD mood episodes when compared to healthy subjects [29]. 

In addition, previous studies suggest that neuroinflammation plays a role in recurrent mood disorders [30,31]. It has been shown that mood episodes can lead to neuronal injury causing the release of DAMPs that in turn activate the microglia [32]. After multiple mood episodes, the excessive release of proinflammatory cytokines can inhibit the neurogenesis and may continue to activate microglial cells, which can lead to pruning and remodeling synaptic plasticity and, ultimately, neuronal death [32]. Microglial and astroglial activation [33], with a consequent reduction in glial density [34] and in the turnover of oligodendrocytes [35,36], have been reported in postmortem studies of BD, which potentially can lead to an increase in the permeability of the blood–brain barrier (BBB) [37]. Of note, microglial activation has been shown in the hippocampus of patients with BD [38]. Moreover, neuroimaging studies have supported the presence of white matter alterations in BD, indicating the involvement of oligodendrocytes [39]. Taken together, these changes support a potential link between neuroinflammation and peripheral inflammation in BD.

It is also important to note that increased levels of cytokines can lead to changes in the production and release of neurotransmitters in the brain [40,41,42]. In this context, changes in monoamine neurotransmitters, such as dopamine, norepinephrine, or serotonin, have been consistently reported in BD and are associated with the mechanisms of action of antidepressants [43]. Altogether, proinflammatory cytokines are associated with reduced synthesis of serotonin and increased neurotoxic metabolites of the kynurenine pathway [44,45,46], which may play a key role in the development of mood symptoms [40,45]. Studies have also shown that increased dopamine neurotransmission is associated with manic symptoms, while decreased dopamine neurotransmission is associated with depressive symptoms [47].

## 3. Impaired Cellular Resilience

Impaired cellular resilience may be one potential explanation for an increased vulnerability to stressful events and episode recurrence among individuals with BD. At the cellular level, resilience is the ability of cells to adapt to different insults or stressful conditions, activating molecular pathways to restore the cellular balance. There is evidence that some patients with BD may present impairments in neuroplasticity and cellular resilience [48] potentially related to the pathological brain rewiring that has been associated with neuroprogression [49]. In addition, increased susceptibility to cell death in the olfactory neuroepithelium [50] and blood cells has been reported in patients with BD [51]. Therefore, the impaired cell resilience reported in BD, both in neurons and peripheral cells, may be associated with an increased vulnerability to cellular stress.

However, the mechanisms underlying the impaired neuroplasticity and cellular resilience in BD have not been fully clarified. The mechanisms causing this reduced resilience most likely involve specific organelles typically responsible for cellular homeostasis such as the mitochondria and the endoplasmic reticulum (ER). Factors including inflammatory signals, oxidative stress, calcium homeostasis disturbances, and other cellular stressful stimuli can disrupt ER and mitochondrial function. It is known that ER and mitochondria are subcellular compartments with a close physical and functional interaction that is crucial for cellular homeostasis and proper functioning [52]. However, disturbances in these organelles under pathological conditions may contribute to impairments in the neural tissue. For instance, research from the past few decades reported apoptotic crosstalk between ER and mitochondria [53], in which ER dysfunction could induce mitochondrial cytochrome-c release and caspase 3 activation leading to apoptosis. In addition, recent findings showed that ER chaperones can control mitochondrial apoptosis through the control of ER–mitochondria calcium flux [54].

Apoptosis has been reported in BD with studies also showing a significant increase in the levels of proapoptotic factors and a decrease in the levels of antiapoptotic factors in both peripheral blood cells [55,56,57] and postmortem brain tissue [58,59]. This may contribute to structural changes in the brain and progressive cognitive changes associated with neuroprogression in BD. Neuroprogression in BD could be an outcome of impaired activation of inflammatory, oxidative, and mitochondrial–ER pathways, which may further lead to impaired neuroplasticity, dysfunction in neuronal signaling, and consequent apoptosis and/or cell death.

## 4. Impaired Calcium Signaling

Research from decades ago found increased levels of calcium in platelets, lymphocytes, and lymphoblastoid cells from patients with BD [60,61]. Among the molecular pathways related to BD, calcium signaling has been highlighted [62,63], supporting previous findings on aberrant calcium signaling in BD [64,65]. In addition, a recent meta-analysis showed that there is a robust increase of basal and stimulated free intracellular calcium in BD [66], reinforcing the role of impaired calcium signaling in this illness. A chronic accumulation of calcium could be associated with cellular dysfunctions and the aforementioned impaired cellular resilience in BD.

Genetic factors may contribute to impaired intracellular calcium signaling in BD. For instance, the genome-wide association study (GWAS) analysis has shown an association of BD with the CACNA1C gene, encoding the α1C subunit of the voltage-gated calcium channel [67]. Whole-genome sequencing analysis also indicated a potential role of calcium signaling-related genes [68,69] in BD. In this scenario, polymorphisms and deletions in mitochondrial DNA have been suggested to affect intracellular calcium regulation in BD [70], supporting the hypothesis of mitochondrial dysfunction and impaired calcium signaling in BD. 

Calcium signaling plays a key role in regulating neuronal excitability and synaptic plasticity involved in cognitive processes. Alterations in this pathway, including abnormally increased calcium levels or changes in properties of calcium channels such as that encoded by the CACNA1C gene, might influence the neuronal membrane excitability, and a phenotypic switch in the excitatory or inhibitory neurons [64]. This could affect essential neural components that could contribute to BD [64]. Calcium activates numerous calcium-dependent enzymes, including kinases, phosphatases, and proteases, and also ion channels [65]. For instance, changes in calcium levels in BD may also act through persistent calcium-dependent protein kinase activation influencing neural circuitry [64]. Recently, more than a hundred calcium-dependent/activated proteins have been related to neuropsychiatry [71], indicating the potential role of calcium signaling in the regulation of oligodendrogenesis mechanisms and myelination involving neural cells and stem/progenitor cells, which are processes potentially involved in BD [39].

Regarding calcium regulation in BD, a recent study that evaluated components of an integrated hormonal system involving parathyroid hormone and vitamin D suggested that chronic calcium imbalance may influence the long-term outcome of BD in terms of clinical severity [72].

## 5. Mitochondrial Dysfunction

Chronic stress and exposure to glucocorticoids have been shown to cause mitochondrial dysfunction, with alterations in oxygen consumption, mitochondrial membrane potential, and calcium holding capacity, ultimately leading to apoptosis [73,74]. Importantly, studies have reported an impaired hypothalamic–pituitary–adrenal (HPA) axis [75], mitochondrial dysfunction [76], and increased apoptosis in patients with BD [55,56,59]. Mitochondrial dysfunction in BD has been evidenced by decreased levels of mitochondrial respiration, high energy phosphates, and pH; changes in mitochondrial morphology; deletions and polymorphisms in mitochondrial DNA; and downregulation of mRNA and proteins involved in mitochondrial metabolism (as reviewed by [70,76]). For instance, postmortem studies have shown decreased expression of genes encoding subunits of complexes of the mitochondrial electron transport chain [77,78,79].

In addition, a recent study evaluating neurons generated from induced pluripotent stem cells (iPSCs) of patients with BD showed neuronal hyperexcitability associated with increased mitochondrial membrane potential, reduced size of mitochondria, and upregulation of mitochondrial genes [80]. Mitochondrial dysfunction in BD is further supported by impaired brain energy metabolism, with metabolic studies reporting increased levels of lactate and gamma-aminobutyric acid in the brain [81] and increased levels of isocitrate in cerebrospinal fluid [82].

These changes in mitochondrial functioning may be associated with impaired calcium signaling and support a key role of this organelle in reduced neuroplasticity underlying neuroprogression in BD. Impaired mitochondrial functioning could lead to an increase in free radicals, causing an imbalance between oxidants and antioxidant mechanisms, potentially resulting in oxidative damage.

## 6. Oxidative Stress

The excessive production of reactive oxygen species (ROS) is the result of an imbalance of oxidant processes and antioxidant defenses, which is called oxidative stress [83]. Oxidative stress has been associated with BD and is closely connected to increased inflammation [84,85]. The increase in ROS can lead to increased damage to lipids, proteins, and DNA, ultimately leading to cell death [83]. Evidence of increased ROS and reduced antioxidant defenses has been reported in patients with BD and is associated with their number of episodes [86,87].

Evidence has pointed to oxidative damage in proteins in BD, such as increased 3-nitrotyrosine [88] and protein carbonyl content [89]. Most findings have focused on patients at late stages, including increased levels of thiobarbituric acid reactive substances (TBARS) [90,91], glutathione reductase, glutathione S-transferase [88], nitric oxide [90], and total oxidant status [92].

## 7. Endoplasmic Reticulum Dysfunction

Evidence from the past two decades has suggested an association between the ER and BD, including pharmacological experiments showing that ER-related chaperones are modulated by mood stabilizers [93,94,95]. Similarly, genetic studies showed an association of BD with a polymorphism in the promoter region of X-box binding protein (XBP)1 (a transcription factor that induces the expression of ER chaperones) [96] and in the chaperone glucose-regulated protein (GRP)78 [97], suggesting them as potential risk factors for developing the disorder. Moreover, lymphoblastoid cells from patients with BD presented a decreased response involving XBP1 and C/EBP homologous protein (CHOP) when exposed to ER stress inducers [98] and a reduction in stress-induced splicing of XBP1 and GRP94 expression [99]. These findings suggest that patients with BD may present with a dysfunctional response to ER stress, which may impair cellular homeostasis.

Interestingly, lymphocytes from patients with BD at late stages presented an impaired response to in vitro induced ER stress (no induction of essential proteins involved in ER stress response signaling) and a pronounced increase in ER stress-induced cell death when compared to patients at early stages and controls [51]. These findings indicate that dysfunction in ER-related stress response may be associated with decreased cellular resilience in BD and that protective cellular mechanisms may become less effective throughout illness progression.

## 8. Biological Basis of Neuroprogression

The knowledge about the biological mechanisms of neuroprogression has advanced considerably in the field of psychiatry, however little is known about the interaction between molecular pathways that may be responsible for clinical outcomes. In addition, it is not known why only a subset of patients may develop a progressive course and worse clinical outcomes, such as reduced responsiveness to treatment, recurrent mood episodes, and impaired cognition and functioning. Understanding such heterogeneity at the biological level may provide valuable information to the field, ultimately providing knowledge related to heterogeneous clinical presentations in patients.

As discussed earlier, increased inflammation is associated with neuroprogression and may be implicated in cellular vulnerability most likely related to a cascade of alterations in mitochondrial function and ER signaling pathways. The ER and mitochondria are tightly interconnected in specific and dynamic microdomains called mitochondria-associated membranes (MAMs). MAMs provide a means of crosstalk between the ER and mitochondria, providing a rapid exchange of physical stimuli and molecules including calcium and ROS [67]. MAMs play crucial roles in calcium signaling, lipid homeostasis, mitochondrial dynamics, autophagy, and inflammation [100]. Disturbances in ER–mitochondria interactions have been associated with the progression of neurological disorders [101]. Herein, we postulate that dysfunctions of the signaling pathway involving these organelles may be important components of illness progression in BD. Considering that (1) BD has been strongly associated with a dysregulation of immune responses, comprising inflammation, oxidative stress, and release of DAMPs, and (2) patients with BD present comorbidities associated with chronic inflammation such as cardiovascular and metabolic diseases, we suggest that the inflammation/MAMs dynamics are the most probable mechanisms linking the systemic changes and impaired cellular resilience observed in patients with BD.

**Figure 2:**
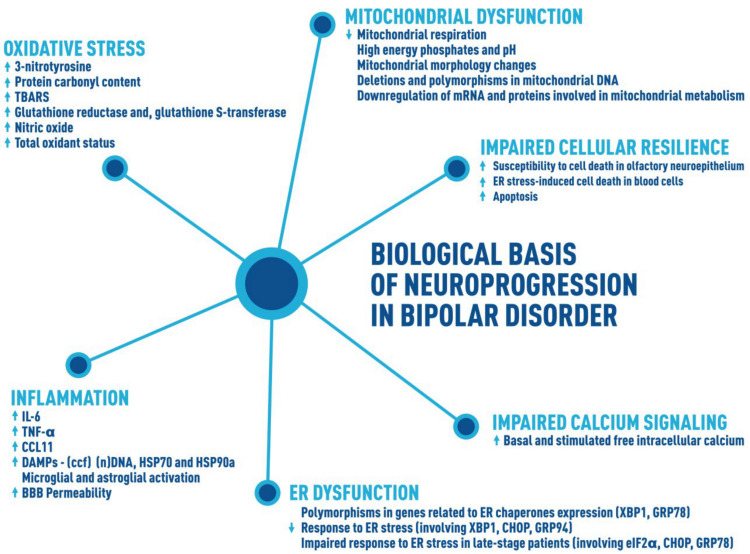
Biological basis of neuroprogression in BD. Several biological changes have been associated with the illness progression in BD. A summary of the main biological pathways that we propose are connected in the neuroprogression of BD is as follows: (1) Inflammation, which includes increased levels of cytokines such as IL-6, TNF-a, and CCL11; increased levels of DAMPs (ccf, (n)DNA, HSP70, and HSP90a); and microglial and astroglial activation, which ultimately can lead to an increase in the permeability of BBB. (2) Oxidative stress, including increased levels of several markers, 3-nitrotyrosine, protein carbonyl content, TBARS, glutathione reductase and glutathione S-transferase, nitric oxide, and total oxidant status. (3) Mitochondrial dysfunction, highlighting the decreased mitochondrial respiration, high energy phosphates, and pH; mitochondrial morphology changes; deletions and polymorphisms in mitochondrial DNA; and downregulation of mRNA and proteins involved in mitochondrial metabolism. (4) ER dysfunction, including polymorphism in genes related to ER chaperone expression (XBP1, GRP78), decreased response to ER stress (involving XBP1, CHOP, GRP94), and impaired response to ER stress in late-stage patients (involving eIF2a, CHOP, GRP78). (5) Impaired calcium signaling, which includes strong evidence showing increased basal and stimulated free intracellular calcium in cells from patients with BD. (6) Impaired neuroplasticity and resilience, represented by the increased susceptibility to cell death and apoptosis. These factors connected may worsen the patient’s clinical outcomes, cognition, and functioning and promote more pronounced changes in peripheral biomarkers and the brain, leading to a progressive illness course (neuroprogression). BD = bipolar disorder, IL = interleukin, TNF = tumour necrosis factor, CCL11 = C-C motif ligand 11, DAMPs = damage-associated molecular patterns, ccf = circulating cell-free, HSP70 = 70 kilodalton heat shock proteins, HSP90a = heat shock 90 kDa protein alpha, BBB = blood–brain barrier, TBARS = thiobarbituric acid reactive substances, ER = endoplasmic reticulum, XBP1 = X-box binding protein 1, CHOP = C/EBP homologous protein, GRP94 = glucose-regulated protein 94, eIF2α = eukaryotic initiation factor 2α, GRP78 = glucose-regulated protein 78.

Thus, we suggest that impairments of cellular resilience may underlie the pathological brain rewiring in BD, contributing to the systemic impairments observed among these patients. In view of that, we propose that the following pathways are interconnected and contribute to neuroprogression in BD: inflammation, oxidative stress, ER and mitochondrial dysfunction, impaired calcium signaling, and impaired neuroplasticity and resilience (Figure 2). These players, interconnected in a vicious loop, may worsen clinical outcomes, cognition, and functioning and promote more pronounced changes in peripheral biomarkers and the brain.

Considering that peripheral markers related to oxidative stress, inflammation, and neurotrophins are altered in patients with BD [26], studies with peripheral samples may yield promising findings in the assessment of neuroprogression pathways in BD. Neuroinflammation appears to orchestrate biological mechanisms that are fundamental to disorders associated with cognitive impairment [102].

## 9. Conclusions

A subset of patients with BD present a neuroprogressive course associated with clinical, functional, cognitive, and biological changes. Molecular pathways and biological markers related to BD have been identified and may help understand the progressive clinical and biological changes associated with this disorder.

We propose that the following pathways are connected in the neuroprogression of BD: (1) inflammation, (2) oxidative stress, (3) impaired calcium signaling, (4) ER dysfunction, (5) mitochondrial dysfunction, and (6) impaired neuroplasticity and cellular resilience. Understanding these pathways may help to clarify the pathophysiology underlying BD and provide novel insights into key molecular players that could be pharmacologically modulated to prevent illness progression, ultimately promoting better outcomes.
